# The *Plasmodium falciparum* homolog of Vps16 interacts with the core members of the Vps-C tethering complex

**DOI:** 10.1128/msphere.00287-25

**Published:** 2025-07-08

**Authors:** Florian Lauruol, Thomas Galaup, Alexandra Bourgeois, Audrey Sergerie, Dave Richard

**Affiliations:** 1 Centre de Recherche en Infectiologie, CRCHU de Québec-Université Laval 177454, Québec City, Canada; 2 Department of Microbiology-Infectious Diseases and Immunology, Faculty of Medicine, Laval University 12369, Québec City, Canada; Weill Cornell Medicine, New York, New York, USA

**Keywords:** malaria, protein trafficking, rhoptries, micronemes, tethering complex

## Abstract

The organelles of the apical complex (rhoptries, micronemes, and dense granules) are critical for erythrocyte invasion by the malaria parasite *Plasmodium falciparum*. Though they have essential roles in the parasite lifecycle, the mechanisms behind their biogenesis are still poorly defined. The Class C Vps proteins Vps11, Vps16, Vps18, and Vps33 constitute the core of the CORVET and HOPS complexes implicated in vesicle tethering and fusion in the eukaryotic endolysosomal system. Work in the model apicomplexan *Toxoplasma gondii* has revealed that TgVps11 is essential for the generation of the apical complex. *P. falciparum* possesses all four subunits of the Vps-C complex, and recent work has shown that some of its components were critical for host-cell cytosol trafficking and the biogenesis of the apical complex. We here show that the *P. falciparum* ortholog of Vps16, a member of the Vps-C complex, is expressed throughout the asexual erythrocytic cycle and that it is potentially associated with the Golgi apparatus and the rhoptries in schizont stage parasites. We then demonstrate by immunoprecipitation and mass spectrometry that PfVps16 interacts with all the members of the canonical Vps-C complex along with the Vps3 CORVET component. Interestingly, three uncharacterized *Plasmodium*-specific proteins are also found as interactors of PfVps16, and structural predictions revealed that two of them possess folds commonly found in proteins present in membrane tethering complexes. These findings suggest that *P. falciparum* may possess both conserved and parasite-specific features within its endosomal tethering machinery.

**IMPORTANCE:**

The malaria parasite relies on special compartments to invade red blood cells. These are key to the parasite’s ability to infect, but how these are generated is not well known. In eukaryotic cells, certain protein assemblies, called tethering complexes, help move and fuse small transport vesicles, which is important for building and maintaining organelles. *Plasmodium falciparum* possesses some of these proteins, and recent studies suggest they play an important role in building its infection machinery and transporting material inside the parasite. We found that the malaria parasite possesses additional components associated with the typical tethering proteins and that these are not found in other eukaryotes. These results suggest that *P. falciparum* uses both common and unique tools to create the cellular machinery it needs to infect red blood cells. We propose that the *Plasmodium*-specific components might represent interesting targets for the development of antimalarials with potentially reduced side effects since they are not present in humans.

## INTRODUCTION

Although significant strides have been made in decreasing the death and illness rates associated with malaria in recent years, the disease continues to impose a substantial burden on various tropical and sub-tropical regions worldwide. In 2023 alone, an estimated 263 million cases causing over 597,000 deaths were attributed to *Plasmodium* parasites, with a majority caused by *Plasmodium falciparum*, the species responsible for the most severe form of the disease (1). The emergence of resistance to many existing antimalarial drugs, including the primary treatment artemisinin (2, 3), underscores the urgent necessity for the development of new intervention approaches.

*Plasmodium* species are obligate intracellular parasites that begin their lifecycle by invading host cells. The invasion of erythrocytes by malaria merozoites involves a complex, multistep process driven by the sequential release of organelles that make up the apical complex: rhoptries, micronemes, and dense granules (4). These organelles are newly formed during a unique cell division process called schizogony (5, 6). Work on intracellular protein trafficking in the model apicomplexan *Toxoplasma gondii* has led to the idea that its endosomal system has been evolutionarily repurposed for rhoptry and microneme formation (7, 8). The mechanisms behind the formation of the apical complex in *P. falciparum* are not as well understood. Conditional knockdown of the Golgi-resident escort protein PfSortilin (9, 10) has shown that it is required for the trafficking of proteins to the micronemes, rhoptries, and dense granules (11). The absence of the Rhoptry apical membrane antigen (RAMA) leads to dysmorphic rhoptry neck, highlighting its importance in their proper biogenesis (12). The cytosolically exposed rhoptry-leaflet-interacting proteins 1 and 2 (CERLI1 and 2) localize to the outside of the rhoptry membrane, and their absence alters the distribution of rhoptry proteins, indicating that they are likely to also play a role in their formation (13–15). Intriguingly, recent work has shown that conditionally knocking down the *P. falciparum* Rhoptry Neck protein 11 (PfRON11) leads to merozoites with a single rhoptry instead of a rhoptry pair (16). Furthermore, *P. berghei* sporozoites where PbRON11 is conditionally knocked down by promoter swap have aberrant rhoptries (preprint (17)), highlighting that RON11 is critical for proper rhoptry biogenesis in at least two species of *Plasmodium* parasites and at different stages of the lifecycle. In *T. gondii*, an intermediate endosome-like compartment (ELC) is involved in protein sorting between the Golgi apparatus and the apical organelles (18), but evidence of a direct pathway from the Golgi apparatus in *P. falciparum* suggests that the ELC may not be necessary in this organism (9). Partial colocalization of the *P. falciparum* homologs of the small G-protein Rab11A and Adaptor protein 1 with rhoptry markers has led to the suggestion that these proteins might play a role in the process of vesicular fusion at the rhoptry membrane (19, 20).

**Fig 1:**
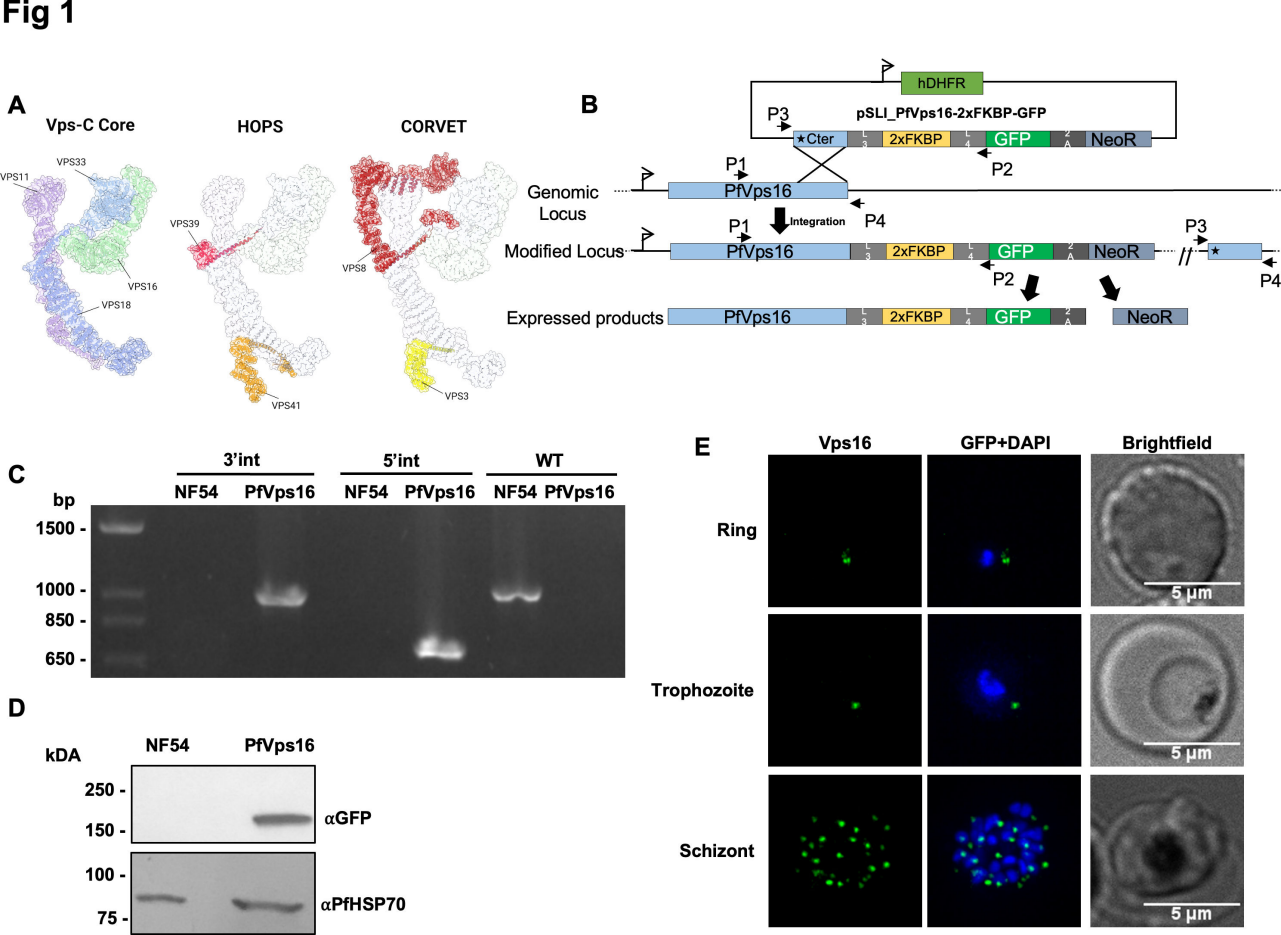
PfVps16 is expressed throughout the asexual erythrocytic cycle. (**A**) 3D representation of the yeast HOPS (PDB: 7ZU0) and CORVET (PDB: 8QX8) complexes. The Vps-C core, composed of VPS11, VPS16, VPS18, and VPS33, is common to the HOPS complex and the CORVET complex. (**B**) Schematic showing the tagging strategy by single cross-over recombination using SLI. (**C**) PCR on parasite genomic DNA showing the proper integration of the tagging vector at the PfVps16 locus (5′ junction: primers P1 and P2, 3′ junction: primers P3 and P4) and the disappearance of the WT allele in the PfVps16-2xFKBP-GFP line (primers P1 and P4). (**D**) Western blot on parasite protein extracts showing the expression of PfVps16-2xFKBP-GFP at the expected size. PfHSP70 is used as a control. (**E**) PfVps16-2xFKBP-GFP is expressed throughout the asexual erythrocytic cycle. Scale bar represents 5 µm. Blue: DAPI-stained nucleus. PfVps16: PfVps16-2xFKBP-GFP.

Eukaryotic cells possess several tethering complexes that capture intracellular vesicles to initiate fusion to their target membrane (21). These complexes are found at distinct subcellular locations and are key to providing specificity (22, 23). CORVET (class C core vacuole/endosome tethering) and HOPS (homotypic fusion and vacuole protein sorting) are tethering complexes associated with the early and late endosomes, respectively, where they play critical roles in processes such as the biogenesis of lysosomes and autophagy (24). They share a conserved core of four proteins, Vps11, Vps16, Vps18, and Vps33, referred to as the Vps-C core (25) and, in yeast and mammalian cells, two specific subunits each (Vps3 and Vps8 for CORVET; Vps39 and Vps41 for HOPS) (26–28) (cartoon representation in Fig. 1A). The Vps-C core of both CORVET and HOPS shares strong structural homology, while the specific subunits are markedly different (29, 30). Intriguingly, the Vps-C core proteins are found in *P. falciparum;* however, only the CORVET-specific Vps3 is conserved, and no Vps8 or either of the HOPS-specific subunits are detectable (25, 31), suggesting specific adaptations of its endolysosomal system. In *T. gondii*, conditional knockdown of TgVps11 and TgVps18 led to the disruption of the biogenesis of the rhoptries, dense granules, and a subset of micronemes (32), and recent work has shown that components of the Vps-C core are implicated in the trafficking of host-cell cytosol and the generation of the apical complex in *P. falciparum* (33).

In this study, we demonstrate that the *P. falciparum* ortholog of Vps16, a component of the Vps-C complex, is expressed throughout the asexual erythrocytic cycle. In schizont-stage parasites, PfVps16 appears to localize to both the Golgi apparatus and the rhoptries. Using immunoprecipitation followed by mass spectrometry, we show that PfVps16 interacts with all canonical components of the Vps-C complex, as well as with the CORVET-specific protein Vps3. Notably, three previously uncharacterized, *Plasmodium*-specific proteins were also identified as interactors; structural predictions suggest that some of these proteins share common folds with those found in membrane tethering complexes, suggesting that they might represent parasite-specific adaptations.

## RESULTS AND DISCUSSION

### Subcellular localization of PfVps16

To explore the role of PfVps16 in the erythrocytic stages, we tagged the endogenous gene at the 3′ end with GFP and a double FK506 binding protein domain (2xFKBP) to allow its functional analysis by knock-sideways (34), by single cross-over recombination using the selection-linked integration (SLI) strategy (34). This line will be referred to as PfVps16-2xFKBP-GFP (Fig. 1B). PCR analysis of genomic DNA from a clonal line revealed proper 5′ and 3′ integration of the plasmid at the *Vps16* locus and the absence of a wild-type (WT) allele (Fig. 1C). Western blots on mixed-stages parasite extracts of the clonal line using an anti-GFP antibody revealed a specific single band at the expected size of around 170 kDa for the PfVps16-2xFKBP-GFP fusion protein. Antibodies against the constitutive protein HSP70 were used as loading controls (Fig. 1D). To determine the expression profile of PfVps16-2xFKBP-GFP throughout the erythrocytic cycle, we performed fluorescence microscopy on tightly synchronized parasites taken at different stages of the cycle. This revealed that the protein was detectable as a single focus in rings, one or more foci in trophozoites, and multiple foci in schizonts (Fig. 1E).

**Fig 2:**
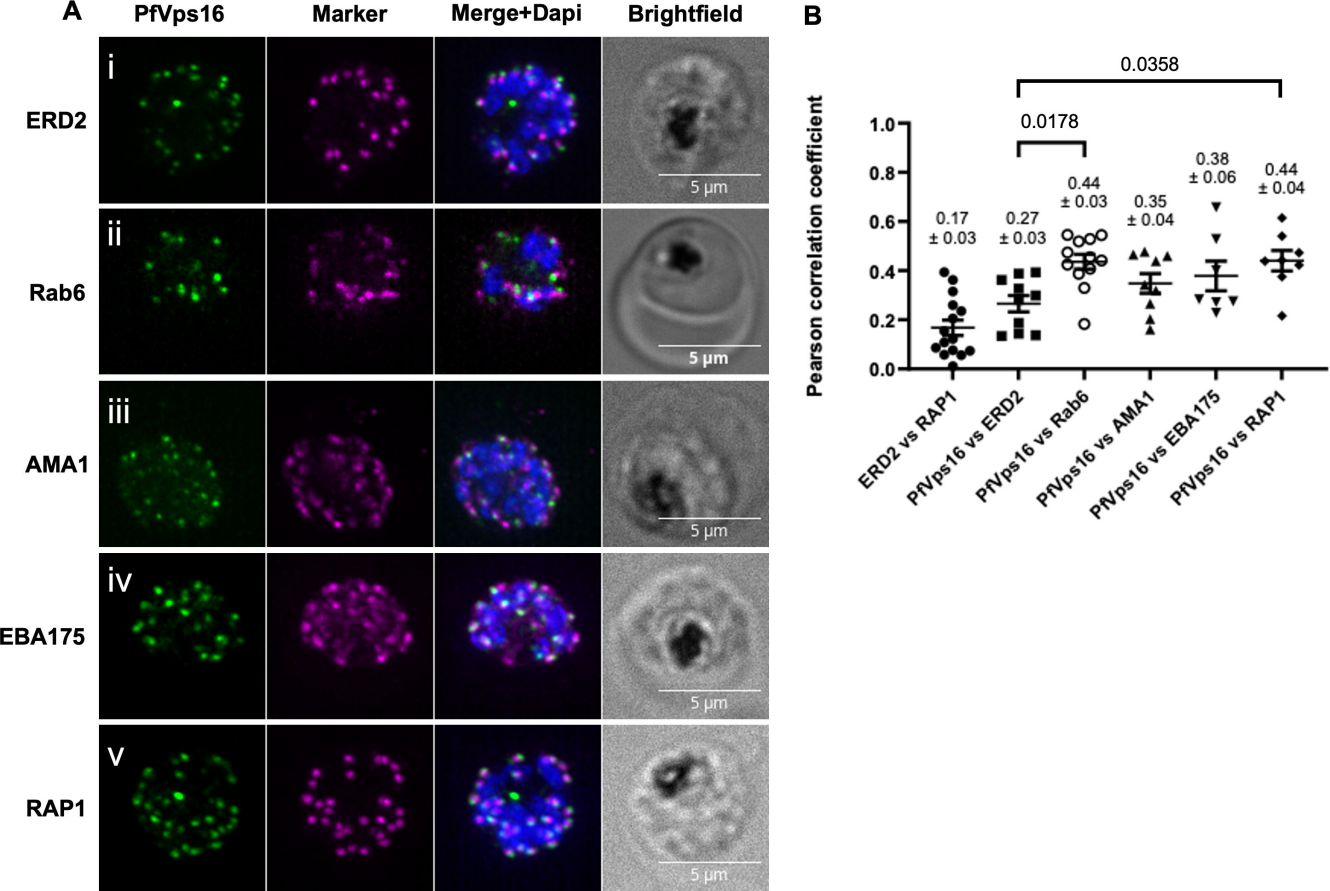
Colocalization analysis of PfVps16. IFA on schizont stage parasites to determine the overlap between PfVps16-2xFKBP-GFP and: (Ai) the cis-Golgi marker ERD2; (Aii) the trans-Golgi marker Rab6; the micronemes markers (Aiii) AMA1 and (Aiv) EBA175; and (Av) the rhoptry marker RAP1. (**B**) Pearson’s correlation analysis demonstrates that PfVps16 overlaps significantly more with Rab6 and RAP1 than with ERD2. ERD2 vs RAP1, *n* = 15, used as a negative control. PfVps16-2xFKBP-GFP vs ERD2, *n* = 10; PfVps16-2xFKBP-GFP vs Rab6, *n* = 12; PfVps16-2xFKBP-GFP vs AMA1, *n* = 9; PfVps16-2xFKBP-GFP vs EBA175, *n* = 7; PfVps16-2xFKBP-GFP vs RAP1, *n* = 8. Each data point represents a whole schizont. Scale bar represents 5 µm. Blue: DAPI-stained nucleus. PfVps16: PfVps16-2xFKBP-GFP. Values represent the mean ± standard error. *P* values were calculated using one-way ANOVA followed by a Tukey’s multiple comparison test. Only the statistically significant *P* values are shown. ERD2 vs RAP1 was not included in the statistical analysis.

We next looked at the subcellular distribution of PfVps16-2xFKBP-GFP by immunofluorescence assays (IFAs) in schizont stage parasites. The pattern of fluorescence being reminiscent of the behavior of proteins found at the Golgi apparatus (9, 10, 35–39), we first determined whether PfVps16-2xFKBP-GFP colocalized with the cis-Golgi marker ERD2. Interestingly, though the foci were often close, there was almost never any overlap (Fig. 2Ai), which suggests that PfVps16-2xFKBP-GFP does not reside at the cis-Golgi. However, strong overlap was observed with the trans-Golgi marker Rab6 (Fig. 2Aii). We next looked at the microneme markers AMA1 and EBA175, which are known to reside in different populations of these organelles (40–42). As seen with ERD2, minimal overlap was observed between the AMA1 and PfVps16-2xFKBP-GFP foci (Fig. 2Aiii). However, some EBA-175 and PfVps16-2xFKBP-GFP foci partially overlapped, whereas others showed no colocalization (Fig. 2Aiv). We finally looked at RAP1, a marker of the rhoptry organelle, and saw that several foci were in very close juxtaposition, while others seemed to strongly overlap (Fig. 2Av). Quantification of the level of colocalization revealed that PfVps16-2xFKBP-GFP overlapped more with Rab6 and RAP1 than with ERD2 (R coefficient of 0.44 ± 0.03 for PfVps16-2xFKBP-GFP vs Rab6; 0.44 ± 0.04 vs RAP1, compared to 0.27 ± 0.03 for PfVps16-2xFKBP-GFP vs ERD2). AMA1 and EBA175 also had higher coefficients than ERD2, but the difference was not statistically significant (0.35 ± 0.04 vs AMA1 and 0.27 ± 0.03 vs EBA175 compared to 0.27 ± 0.03 for PfVps16-2xFKBP-GFP vs ERD2) (Fig. 2B). The fact that not all foci share the same level of overlap suggests that PfVps16-2xFKBP-GFP is not a resident of the trans-Golgi or the apical organelles but that it might shuttle between them as seen with several proteins of the intracellular trafficking machinery such as PfSortilin (10, 11), PfVps45 (43), and PfRbsn5L (44).

### Attempts to mislocalize PfVps16-2xFKBP-GFP to the nucleus are unsuccessful

To delve into the role of PfVps16 in the asexual blood stages, we attempted to perform knock-sideways, a strategy that allows the conditional removal of a protein of interest from its site of action (45). To do so, we transfected the PfVps16-2xFKBP-GFP parasite line with a plasmid expressing a nuclear mislocalizer consisting of a triple nuclear localization signal fused to a double FKBP12-rapamycin binding domain and the mCherry fluorescent protein (3xNLS-2xFRB-mCherry) (34). We first tested the ability of the mislocalizer to translocate PfVps16-2xFKBP-GFP to the nucleus when rapamycin was added to the medium (Rapa), thus removing it from its normal site of action. In the absence of Rapa, the mislocalizer overlapped with the DAPI-stained nucleus, while PfVps16-2xFKBP-GFP showed its normal punctate pattern (Fig. S1Ai). When adding Rapa at the ring stage and letting the parasites mature to trophozoites and schizonts, GFP remained as foci that did not overlap with the DAPI (Fig. S1Aii and iii). Instead, some of the mCherry signal was now colocalizing with GFP foci (Fig. S1Aii and iii, white arrows). This suggests that not only is PfVps16-2xFKBP-GFP not translocated into the nucleus, but that some of the mislocalizer is actually delocalized from the nucleus by PfVps16-2xFKBP-GFP. This reverse mislocalization has been previously observed (46). This might be potentially explained by a potential strong association of PfVps16-2xFKBP-GFP with the other members of the Vps-C complex preventing its extraction. Interestingly, while we were completing this manuscript, a study came out showing that PfVps16 tagged with the sandwich version of the 2xFKBP-GFP system using 2xFKBP both at the N- and C-terminus of GFP (2xFKBP-GFP-2xFKBP) was efficiently translocated to the nucleus and that this led to parasite death. Further characterization of the phenotype revealed a defect in merozoite invasion of the red blood cell due to a mistrafficking of rhoptry and microneme proteins in schizonts (33).

### PfVps16 interacts with members of the core Vps-C complex

**Fig 3:**
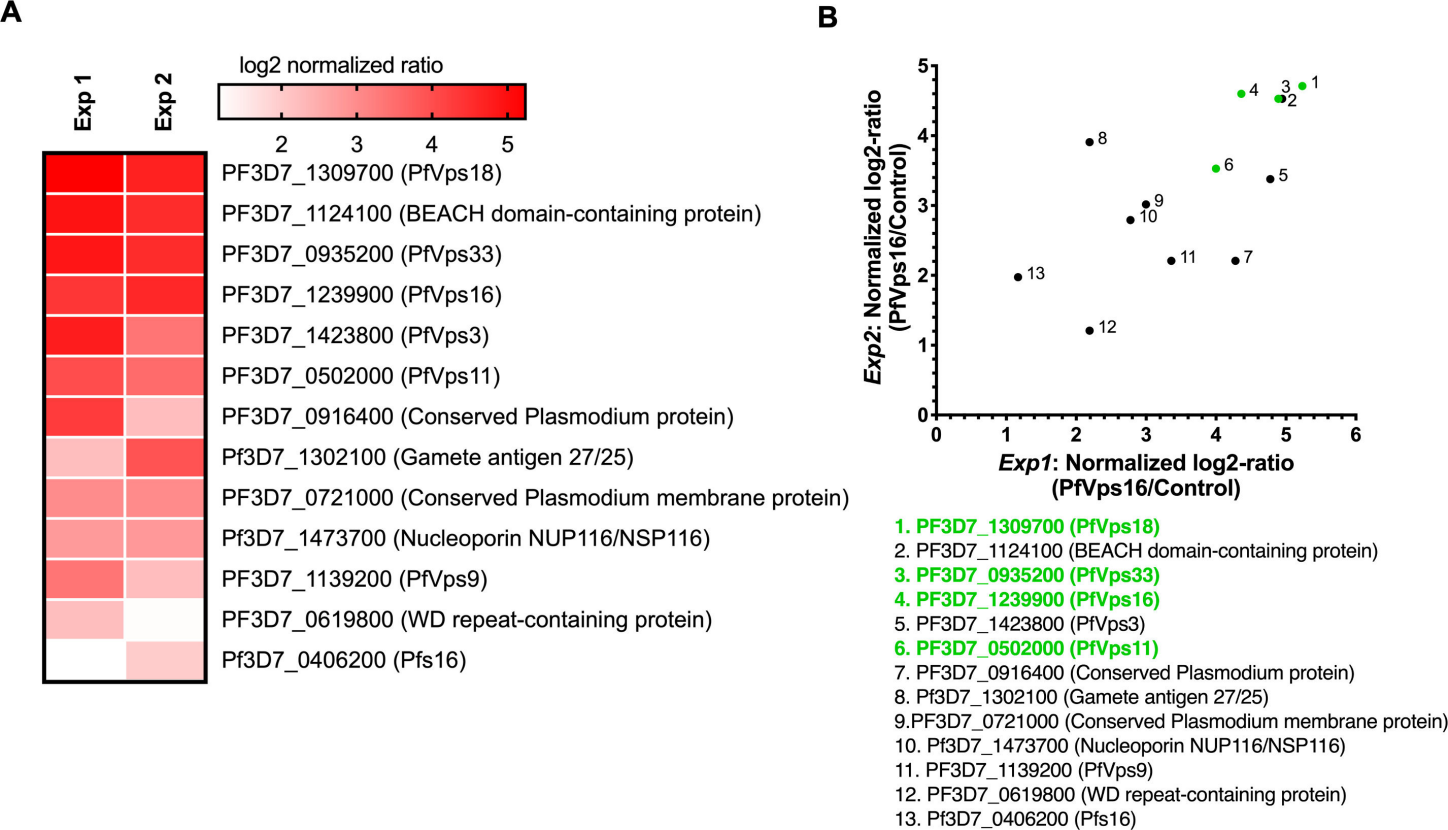
Identification of proteins interacting with PfVps16. (**A**) Heatmap of proteins found enriched in two biological replicates of a PfVps16 immunoprecipitation. Protein enrichment is represented by log2 normalized ratio of PfVps16/Control (WT) for each experiment. PlasmoDB identifiers, as well as descriptive names, are indicated for each enriched protein. (**B**) Scatter plot indicating enriched proteins found in both biological replicates of a PfVps16 immunoprecipitation. Protein enrichment is represented by log2 normalized ratio of PfVps16/Control (WT) for each experiment. PlasmoDB identifiers, as well as descriptive names, are indicated for each enriched protein. Proteins highlighted in green are known members of the Vps-C core. The complete mass spectrometry data are available in Table S1.

We next wanted to determine whether the interaction between the members of the core Vps-C complex was conserved in *P. falciparum*, so we first attempted to use dimerization-induced BioID for proximity-dependent biotinylation (47). However, we were not able to recover parasites after transfecting the PfVps16-2xFKBP-GFP parasite line with a plasmid expressing the biotin ligase BirA in fusion with FRB. We therefore performed standard immunoprecipitations on schizont protein extracts with anti-GFP agarose beads followed by mass spectrometry, which would likely allow us to identify proteins stably interacting with PfVps16-2xFBKP-GFP, such as would be expected for Vps-C core components. An untagged NF54 parasite line was used as a negative control. More than 500 proteins were identified in each of two biological replicates (Table S1), but only 10 proteins were enriched at least twofold over the control in both biological replicates (Fig. 3). As in other eukaryotic cells, our results showed that PfVps16 interacted with all the other core Vps-C members Vps11, Vps18, and Vps33, demonstrating that this complex is indeed conserved in *P. falciparum* blood stages. Vps11 and Vps18 form the central scaffold essential for the assembly of both CORVET and HOPS complexes (29, 30), and its deletion results in endolysosomal defects in yeast (48, 49). Furthermore, mutations/variants in Vps11 have been associated with human diseases such as leukoencephalopathy (50), generalized dystonia (51), and Parkinson’s (52). Importantly, the conditional knockdown of *T. gondii* Vps11 is lethal due to a disruption in the biogenesis of the rhoptries, micronemes, and dense granules (32), and a recent manuscript showed that PfVps11 and PfVps18 were critical for host-cell cytosol delivery to the food vacuole and for the trafficking of rhoptry and microneme proteins (33). Interestingly, though we have here shown that PfVps16 forms a complex with PfVps11 and PfVps18, Mesén-Ramírez et al. have shown that the former is not involved in the trafficking of host-cell cytosol (33). This suggests that different sub-complexes could have different functions.

In addition to members of the Vps-C complex, we identified a putative ortholog of Vps3 (PF3D7_1423800), a member of the CORVET complex. It is interesting to note that *Plasmodium* parasites do not encode recognizable orthologs of the other critical CORVET component Vps8 nor of the HOPS-specific components Vps39 and Vps41 (25, 31). The targeting of the CORVET and HOPS complexes to their specific subcellular locations is done through the interactions of their specific subunits with distinct Rab GTPases (Vps3 and Vps8 with Rab5 on early endosomes (27, 53); Vps39 and Vps41 with Rab7 on late endosomes, autophagosomes, and the yeast vacuole (48, 54)). CryoEM structures of both complexes have shown that each specific subunit is located at distal ends of their respective complexes, potentially each binding Rabs on two opposing membranes, thus facilitating their fusion (29, 30). In the absence of these subunits, it is unclear if the *P. falciparum* complex possesses the canonical tethering function. There is, however, precedence for reduced CORVET complexes since, in Drosophila, Vps3 is absent, and Vps8 forms a miniCORVET complex with Vps18, Vps33A, and Vps16 but devoid of Vps11. This complex is critical for the tethering of early endosomes (25, 55). Intriguingly, contrarily to PfVps11, PfVps16, and PfVps18, the conditional inactivation of PfVps3 was recently shown to only have a minor impact on the proliferation of *P. falciparum,* revealing that the functionalities of the different members of the HOPS/CORVET are quite different from other eukaryotes (33).

Further examination of the putative PfVps-C interactors identified a conserved *Plasmodium* protein (PF3D7_0916400), a WD-repeat containing transmembrane protein (PF3D7_0619800), and a large uncharacterized transmembrane protein (PF3D7_0721000). All three are potentially essential (56), but whether they play roles in the functions of the PfVps-C remains to be determined. We also identified a homolog of the CORVET complex interactor Vps9, a GTP-exchange factor that stimulates the exchange of GDP to GTP in Rab5, resulting in its activation (27, 57). *In T. gondii*, Vps9 localizes to the endosomal-like compartment and is implicated in the generation of micronemes, rhoptries, and dense granules (32, 58). TgVps9 also interacts with the Sortilin-like receptor, a protein that is required for apical secretory organelle biogenesis in both *T. gondii* (59) and *P. falciparum* (10, 11). A BEACH domain-containing protein (BDCP) was also found in the PfVps16 interactome. Homologs of these proteins are conserved in eukaryotes and play roles in processes such as cargo sorting (60), vesicular fusion and fission (61), and autophagy (62). A BDCP was identified as interacting with TgVps11, and its conditional knockdown resulted in alterations in the morphologies of rhoptries and the vacuole (63). Finally, PfNup116, Pfs16, and the Gamete antigen 27/25 were also found as putative interactors but might potentially be contaminants. Pfs16 and Gamete antigen 27/25 are gametocyte proteins, the former localizes to the parasitophorous vacuole membrane (64, 65) whilst the latter is an abundant RNA binding protein (66, 67). PfNup116 is a protein found in the nuclear pore (68). Globally, our data shows that a canonical Vps-C complex is present in *P. falciparum* asexual stages and associates with the CORVET subunit PfVps3, PfVps9, and PfBDCP, the latter two for which the orthologs in *T. gondii* are implicated in the biogenesis of the secretory organelles of the apical complex.

### Analysis of the predicted 3D structure of the putative CORVET components

**Fig 4:**
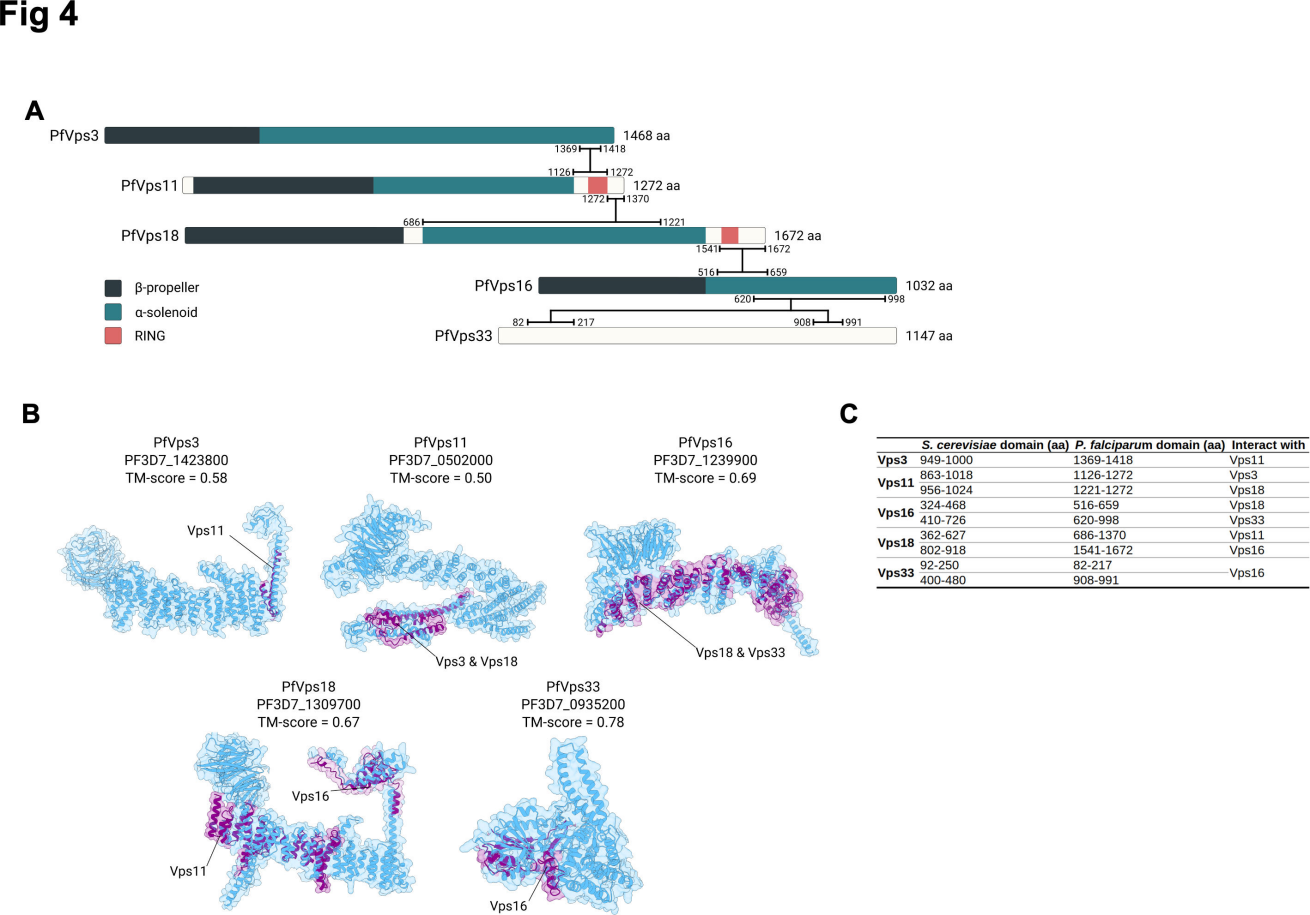
Structural Predictions and Interaction Domains of the CORVET Complex in *P. falciparum*. (**A**) Schematic representation of the predicted interactions between the *P. falciparum* components of the CORVET complex. Domain annotations: dark blue for β-propeller domains, light blue for α-solenoid domains, and salmon for RING domains. Horizontal black lines indicate the predicted regions involved in protein-protein interactions. (**B**) Predicted 3D structures of *P. falciparum* Vps3 and Vps-C proteins (blue) aligned with the experimentally determined interaction domains of *S. cerevisiae* CORVET complex (purple, PDB: 8QX8). Disordered regions in the *P. falciparum* proteins have been omitted for clarity. Black lines indicate the *S. cerevisiae* Vps proteins that interact with the highlighted domains (purple). (**C**) Table comparing the interaction domains of *S. cerevisiae* CORVET complex components with their predicted counterparts in *P. falciparum*. The table lists the amino acid regions mediating interactions between the proteins in both species.

Our immunoprecipitation data indicated that PfVps16 interacted with PfVps3, PfVps11, PfVps18, and PfVps33—proteins known to be components of the CORVET complex. The formation of this complex requires specific interactions between the different Vps proteins via conserved domains (29). To investigate whether the *P. falciparum* putative CORVET components possessed the conserved structural features for proper complex assembly, we predicted their 3D structures using the AlphaFold3 algorithm (69) (Fig. S2). Predictions for PfVps3, PfVps16, and PfVps33 yielded relatively high-confidence models, with predicted template modeling (pTM) scores exceeding 0.50 and the majority of residues displaying a predicted local-distance difference test (pLDDT) score above 50. In contrast, the predicted 3D structures of PfVps11 and PfVps18 were of lower confidence, with pTM scores ranging from 0.40 to 0.50 and multiple disordered regions. These disordered regions correspond to low-complexity regions (LCRs) within the protein sequence, which are prevalent in *P. falciparum* (70)*,* though their functional significance remains debated. Some LCRs, such as D/E repeats, have been implicated in gene regulation (71). AlphaFold3 appears to struggle with predicting the 3D conformation of these LCRs, often representing them as disordered regions with a ribbon-like appearance. However, rather than indicating a failure in structure prediction, these low-pLDDT regions likely reflect inherent protein flexibility or disorder (72). Such regions may adopt specific conformations only upon interacting with binding partners, making their accurate prediction by AlphaFold particularly challenging. We next used InterPro (73) to analyze the domain composition of these proteins and found that, similar to *S. cerevisiae* (29), PfVps3, PfVps11, PfVps16, and PfVps18 possess an N-terminal β-propeller followed by an α-solenoid domain. PfVps11 and PfVps18 also feature a RING (Really Interesting New Gene) domain at their C-terminus (Fig. 4A).

Next, we sought to determine if the specific binding domains mediating the formation of the CORVET complex were present within the *P. falciparum* proteins. To achieve this, we first mapped the interacting domains of *S. cerevisiae* Vps3, Vps11, Vps16, Vps18, and Vps33 proteins using the experimentally determined structure of the CORVET complex (PDB: 8QX8) (59). These domains were then aligned with their predicted counterparts in *P. falciparum* using US-Align (74) (Fig. 4B and C), yielding consistently high TM-scores (all >0.5), indicative of strong structural conservation. This suggests that the five *P. falciparum* CORVET proteins retain key structural features necessary for complex assembly. The predicted interaction domains within the *P. falciparum* CORVET complex are illustrated in Fig. 4A.

**Fig 5:**
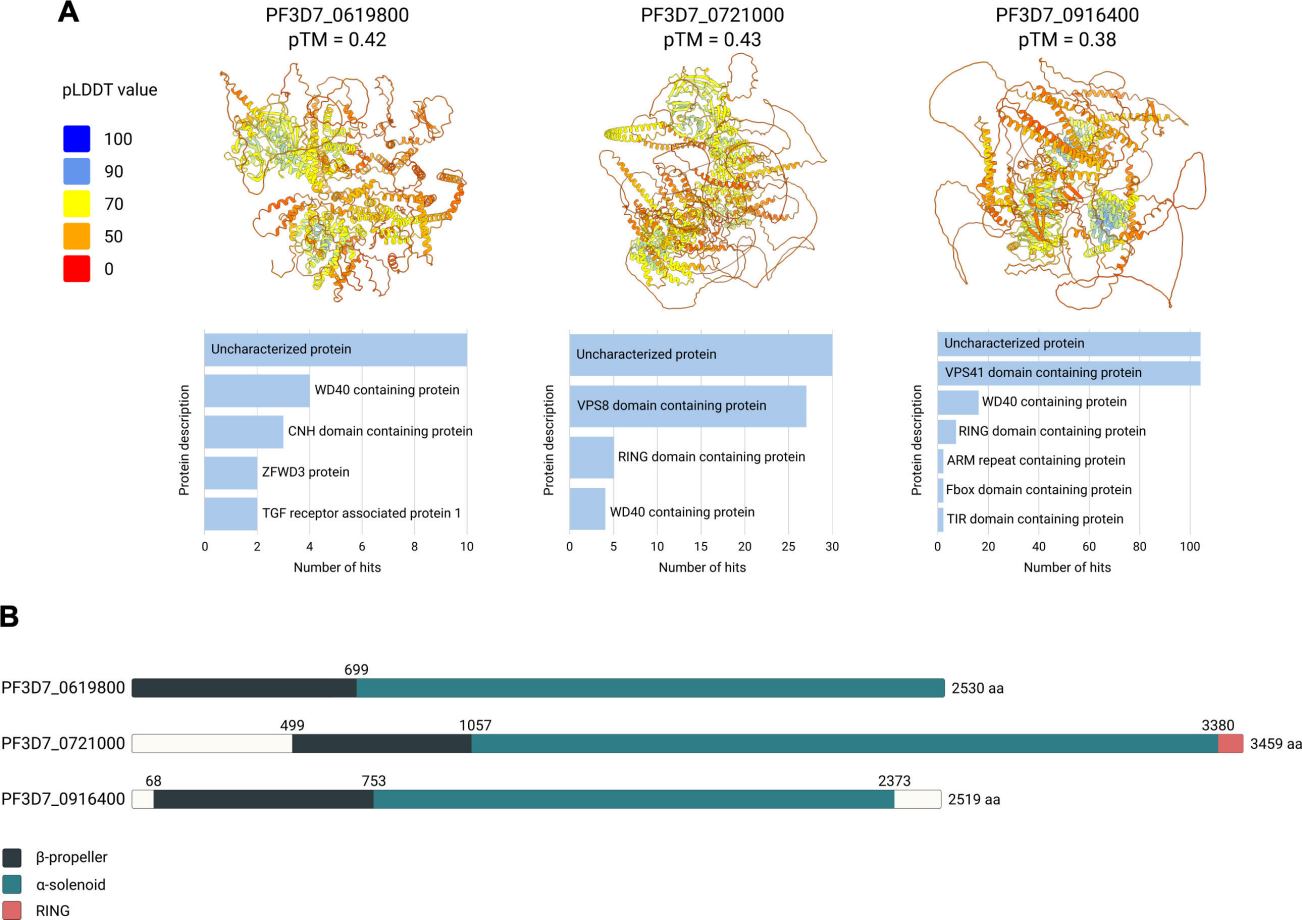
Structural predictions and FoldSeek homology analysis of three uncharacterized *P. falciparum* proteins found in a complex with PfVps16 (**A**) AlphaFold 3 structure prediction of PF3D7_0619800, PF3D7_0721000, and PF3D7_0916400. 3D structures are color coded with predicted local-distance difference test (pLDDT) value. Horizontal bar charts: FoldSeek structural similarity searches using the predicted structures as queries. Hits were grouped by protein description, merging synonymous annotations (e.g., “Vps8,” “Vacuolar-associated protein 8,” and “Vps8 domain containing protein” are all represented as “Vps8 domain containing protein”). Only categories with more than one hit are displayed. (**B**) Schematic representation of the predicted domains inferred from the predicted 3D structures of the proteins. Domain annotations: dark blue for β-propeller domains, light blue for α-solenoid domains, and salmon for RING domains.

Interestingly, co-IP data revealed that PfVps16 interacts with three previously uncharacterized proteins: PF3D7_0619800, PF3D7_0721000, and PF3D7_0916400. We first performed a protein BLAST (75) to identify homologs of these proteins in other species. However, all hits were restricted to *Plasmodium* parasites (Table S2). To gain insight into their potential functions, we predicted their 3D structures using AlphaFold3 (Fig. 5A). The structural predictions for PF3D7_0619800 and PF3D7_0916400 were of moderate confidence (pTM = 0.42 and 0.38, respectively), with extensive disordered regions. Despite the low confidence, their predicted structures display features reminiscent of known CORVET/HOPS proteins, including a N-terminal β-propeller followed by an α-solenoid domain, which are hallmarks of vesicular tethering proteins (76) (Fig. 5B). Among the three, PF3D7_0721000 had a slightly higher structural prediction confidence (pTM = 0.43), with a more discernible “tethering protein-like” organization, consisting of three clearly defined domains: a C-terminal β-propeller, a central α-solenoid, and an N-terminal RING domain.

We next performed a structural similarity search using Foldseek (77). While no significant matches were found for PF3D7_0619800, PF3D7_0721000, and PF3D7_0916400 yielded multiple hits for Vps8 and Vsp41 proteins, respectively, across different species (Fig. 5A; Table S1). Structural alignment of PF3D7_0721000 with *S. cerevisiae* Vps8 (PDB: 8QX8) (29) using US-align (74) resulted in a TM-score of 0.515 across 1,117 residues, indicating moderate structural similarity. Similarly, PF3D7_0916400 aligned with *S. cerevisiae* Vps41 (PDB: 7ZU0) (29) with a TM-score of 0.508. However, this alignment is based on a limited portion of the yeast protein—only 112 amino acids out of a total length of 1,016 are resolved—making it a partial and somewhat constrained structural comparison. Notably, both PF3D7_0721000 and PF3D7_0916400 are considerably longer than their putative *S. cerevisiae* counterparts, with 3,459 and 2,519 amino acids respectively, compared to 1,292 for ScVps8 and 1,016 for ScVps41. This size discrepancy is sometimes found as *P. falciparum* proteins are often characterized by extensive LCRs and unusually long N-terminal extensions. These features are common across the parasite’s proteome and contribute to the apparent divergence in sequence and structure compared to homologs in other eukaryotes (78, 79).

Based on the domain architecture, structural similarity, and observed interactions with PfVps16, we hypothesize that PF3D7_0721000 and PF3D7_0916400 may represent the *P. falciparum* equivalents of Vps8 and Vps41, respectively. Nevertheless, this hypothesis remains speculative, as structural resemblance alone is insufficient to confirm functional equivalence. Experimental validation will be necessary to determine whether these proteins truly function as Vps8 and Vps41 components in the parasite’s vesicular trafficking machinery.

Despite the poor structural predictions and absence of clear homologs, PF3D7_0619800 interacts with PfVps16 and appears to be *Plasmodium*-specific. This raises the possibility that this protein could play a role in the parasite’s endosomal tethering machinery.

### Conclusion

Taken together, we presented evidence that PfVps16 is constitutively expressed throughout the asexual blood stages and that it potentially shuttles between the Golgi apparatus and some of the organelles of the apical complex. Furthermore, we have shown that PfVps16 forms a complex with members of the canonical Vps-C complex and the CORVET-specific subunit Vps3. The predicted structural features and observed interactions of PfVps3, PfVps11, PfVps16, PfVps18, and PfVps33 are consistent with their counterparts in other organisms, supporting the idea of a functionally analogous Vps-C core complex in the parasite. Additionally, structural and interaction data led us to speculate that PF3D7_0721000 and PF3D7_0916400 may perhaps act as functional analogs of Vps8 and Vps41, respectively, though we acknowledge the limited sequence homology and substantial divergence in protein length. The identification of PF3D7_0619800 as a PfVps16 interactor lacking clear homologs in other eukaryotes potentially points to a unique adaptation in the *P. falciparum* vesicular trafficking system. These findings highlight both conserved and parasite-specific features of the endosomal machinery, providing a foundation for future functional studies to elucidate their roles.

## MATERIALS AND METHODS

### Parasite culture

*P. falciparum* NF54 asexual stage parasites were cultured under standard conditions in RPMI-HEPES medium at 4% hematocrit (human erythrocytes of O^+^ group) and 0.5% (wt/vol) Albumax (Invitrogen) and kept at 37°C in a gas mixture of 5.0% oxygen, 5.0% carbon dioxide, and 90% nitrogen (80).

### Vector construction and transfection

To tag the endogenous PfVps16 with 2xFKBP-GFP, around 500 bp of the C-terminus of PfVps16 was amplified with primers 5′ Not1-2571-PfVps16 (ATAgcggccgcGAATAGTCATATAAAATTTGTTCATACTTCC) and 3′ AvrII-stopless-PfVps16 (ATAcctaggTCTTATGTTTGATATGGCATC) and cloned in frame with 2xFKBP-GFP in pSLI-2xFKBP-GFP-hDHFR digested NotI-AvrII (34). Parasites were transfected, and integrants were selected as described previously with some modifications (34). Briefly, *P. falciparum* NF54 parasites were transfected with 100 µg of purified pSLI-PfVps16-2xFKBP-GFP plasmid. Positive selection for transfectants was achieved using 5 nM WR99210 (WR). Then drug-resistant parasites were split into three separate wells with 2–4% parasitemia and went under another round of selection using 400 µg/mL neomycin (NEO) to select for integrants. After parasite re-emergence (after around 10 days), WR was put back in the culture medium. Parasites were then cloned by limiting dilution, resulting in the PfVps16-2xFKBP-GFP line. Genomic DNA was prepared from NEO and WR-resistant parasites. Integration was monitored by PCR using the forward 5′ upstream-2571-PfVps16-F (primer 1, GGGCAACATTCGCAAGC) and the reverse 3′ 90-GFP-R primer (primer 2) for 5′ integration and 5′ pARL-F (primer 3) with the reverse 3′ UTR-PfVps16-R primers (primer 4, GGTGAAAATAGAACTCGATGC) for the 3′ integration. Primer 1 was used with primer 4 to detect the WT version of the gene.

To generate the parasite line for the knock-sideways, the PfVps16-2xFKBP-GFP line was transfected with 100 µg of purified p3xNLS-FRB-mCherry-BSD plasmid (34) and selected with 2 µg/mL blasticidin (Sigma-Aldrich) to obtain the PfVps16-2xFKBP-GFP + mislocalizer line.

### Western blotting

Saponin-extracted parasites from asynchronous PfVps16-2xFKBP-GFP line were harvested. Proteins were then separated on 10% (wt/vol) SDS-PAGE gel under reducing conditions and transferred to a PVDF membrane (Millipore). The membrane was blocked in 4% (wt/vol) milk in TBS-T. Antibodies used were mouse monoclonal anti-GFP 1:500 (ROCHE; IgG clones 7-1 and 13-1) and rabbit polyclonal anti-PfHSP70 1:40,000 (StressMarq Bioscience Inc, SPC-186C) (81). Appropriate HRP-coupled secondary antibodies were used, and immunoblots were developed using ECL (Bio-Rad).

### Microscopy

Fluorescence microscopy acquisition was performed as previously described (38) using a GE Applied Precision Deltavision Elite microscope with a 100× 1.4 NA objective and with a sCMOS camera and deconvolved with the SoftWorx software. For IFAs, parasites were fixed with 4% paraformaldehyde-0.0075% glutaraldehyde (ProSciTech). After blocking in 3% bovine serum albumin (BSA fraction V, EMD), the slides were probed with combinations of antibodies: rabbit anti-ERD2 (MRA-72, 1:1,000) (37); rabbit anti-AMA1, 1:1,000 (82) or mouse monoclonal anti-AMA1 (clone 1F9, 1:500) (83); rabbit anti-PfEBA175 (1:1,000)(84), mouse anti-RAP1 (1:3,000)(85). Primary antibodies were probed with Alexa Fluor 594 anti-rabbit IgG or anti-mouse IgG (Molecular Probes) and Alexa Fluor 488 anti-rabbit IgG or anti-mouse IgG (Cell Signaling). Slides were mounted with 4′,6-Diamidino-2-phenylindole dihydrochloride (DAPI; Invitrogen, 100 ng/µL) in VectaShield (Vector Labs) or ProLong Gold anti-fade (Molecular Probes). Pearson’s correlation coefficients between Alexa488 and Alexa594 channels were calculated on deconvolved regions of interests of image stacks containing a whole schizont, including zero-zero pixels and without thresholding using the SoftWorx software (GE). Data were analyzed for statistical significance using one-way ANOVA followed by a Tukey multiple comparison test. Chromatic calibration of the microscope was performed prior to imaging experiments.

### Immunoprecipitation using anti-GFP agarose beads

Immunoprecipitation using anti-GFP beads was performed using three 30 mL schizont cultures of both PfVps16-2xFKBP-GFP and WT NF54 parasites. Parasite cultures were synchronized twice by using 5% sorbitol treatments, as previously described in reference 86 at a 16 h interval. Afterwards, parasitemia was adjusted to about 7% and parasites were left to grow for 20 h. Following this, parasite cultures were treated with saponin to release host cell cytosol, and pellets were kept at −80°C until immunoprecipitation. Thawed parasite pellets were resuspended in 1% T-Net containing cOmplete EDTA-free protease inhibitor (Roche) and incubated for 1 h with agitation. Samples were then centrifuged for 10 min at 16,000 rpm in order to separate the insoluble and soluble protein fractions. Soluble protein fractions were then transferred to new microcentrifuge tubes containing the pre-washed anti-GFP mAb-agarose beads (MBL) and incubated for 1 h and 30 min with agitation. Beads were then spun down at 500 × *g* for 5 min and washed two times in 1× PBS before being resuspended in 100 µL of 1× PBS and kept at −80°C until mass spectrometry analysis. Protein samples were incubated at 4°C unless otherwise specified. Anti-GFP mAb-agarose beads were then sent for mass spectrometry analysis. Interaction partners were analysed using Scaffold5, and the normalized total spectra graph was produced using GraphPad.

### Sample preparation and data acquisition for mass spectrometry analysis

Protein digestion and mass spectrometry experiments were performed by the Proteomics platform of the CHU de Quebec Research Center, Quebec, Canada

#### Protein digestion

Protein digestion and mass spectrometry experiments were performed by the Proteomics platform of the CHU de Quebec Research Center, Quebec, Canada. On beads, protein digestion was carried out using 0.1 µg of modified porcine trypsin (sequencing grade, Promega, Madison, WI) in 50 mM ammonium bicarbonate for 5 h at 37°C. Digestion was stopped with 5% formic acid (FA), and peptides were eluted from the beads with 60% acetonitrile (ACN) 0.1% FA. Tryptic peptides were desalted on Stage tips (Empore C18, 3M Company), vacuum dried, then resuspended in LC loading solvent (2% ACN, 0.05% trifluoroacetic acid [TFA]).

#### Mass spectrometry

Half of each sample was analyzed by nanoLC/MSMS using a Dionex UltiMate 3000 nanoRSLC chromatography system (Thermo Fisher Scientific, San Jose, CA, USA) connected to an Orbitrap Fusion mass spectrometer (Thermo Fisher Scientific) equipped with a nanoelectrospray ion source. Peptides were trapped at 20 µL/min in loading solvent (2% ACN, 0.05% TFA) on a 5 mm × 300 µm C18 pepmap cartridge (Thermo Fisher Scientific) during 5 min. Then, the pre-column was switched online with a 50 cm × 75 µm internal diameter separation column (Pepmap Acclaim column, ThermoFisher), and the peptides were eluted with a linear gradient from 5% to 40% solvent B (A: 0.1% FA, B: 80% ACN, 0.1% FA) in 30 min, at 300 nL/min (60 min total runtime). Mass spectra were acquired using a data-dependent acquisition mode using Thermo XCalibur software version 4.1.50. Full scan mass spectra (350–1,800 *m/z*) were acquired in the Orbitrap using an AGC target of 4e5, a maximum injection time of 50 ms and a resolution of 120,000. Internal calibration using lock mass on the *m/z* 445.12003 siloxane ion was used. Each MS scan was followed by the acquisition of fragmentation MSMS spectra of the most intense ions for a total cycle time of 3 s (top speed mode). The selected ions were isolated using the quadrupole analyzer with 1.6 *m/z* windows and fragmented by Higher energy Collision-induced Dissociation (HCD) with 35% of collision energy. The resulting fragments were detected by the linear ion trap in rapid scan rate with an AGC target of 1e4 and a maximum injection time of 50 ms. Dynamic exclusion of previously fragmented peptides was set for a period of 30 s and a tolerance of 10 ppm.

#### Database searching

MGF peak list files were created using Proteome Discoverer 2.3 software (Thermo). MGF files were then analyzed using Mascot (Matrix Science, London, UK; version 2.8.0). Mascot was set up to search a contaminant database and Uniprot *Plasmodium Falciparum* 3D7 (5538 entries, reference proteome UP000001450) database assuming the digestion enzyme trypsin. Mascot was searched with a fragment ion mass tolerance of 0.60 Da and a parent ion tolerance of 10.0 PPM. Carbamidomethyl of cysteine was specified in Mascot as a fixed modification. Deamidation of asparagine and glutamine and oxidation of methionine were specified in Mascot as variable modifications. Two missed cleavages were allowed.

#### Criteria for protein identification

Scaffold (version Scaffold_5.1, Proteome Software Inc., Portland, OR) was used to validate MS/MS based peptide and protein identifications. A false discovery rate of 1% was used for peptide and protein. Proteins that contained similar peptides and could not be differentiated based on MS/MS analysis alone were grouped to satisfy the principles of parsimony.

### Knock-sideways attempts

Tightly synchronous ring stage PfVps16-2xFKBP-GFP + mislocalizer parasites were seeded at 2% parasitemia and grown ± 250 nM rapamycin. After 24 and 48 h in culture, the cells were harvested, stained with DAPI (Invitrogen, 100 ng/µL) and imaged immediately.

### Statistical analysis

Prism 7 (GraphPad) was used for all statistical analyses. Depending on the assay, one-way ANOVA or two-tailed unpaired *t*-tests were performed. A *P* value of <0.05 was considered statistically significant.

### Protein structure prediction

The protein sequences of PfVps16 (PF3D7_1239900), PfVps11 (PF3D7_0502000), PfVps16 (PF3D7_1239900), PfVps18 (PF3D7_1309700), PfVps33 (PF3D7_0935200), PF3D7_0619800, PF3D7_0721000, and PF3D7_0916400 were retrieved from the *Plasmodium falciparum* genome database (plasmodb.org). Their 3D structures were predicted using the AlphaFold server (https://alphafoldserver.com), which implements the AlphaFold 3 algorithm (69). All generated files are available at https://doi.org/10.6084/m9.figshare.29225000.v1

### Structural alignment

To identify the interaction domains of Vps proteins within the CORVET complex, we used the experimentally determined 3D structure of the *Saccharomyces cerevisiae* CORVET complex (PDB: 8QX8) (27). The *Interfaces* tool in ChimeraX (version 1.8) (87) was used to isolate amino acid residues involved in protein-protein interactions within the complex. The identified interaction domains were then aligned with their *P. falciparum* Vps homologs using US-align (74) (https://zhanggroup.org/US-align/) (version 20240510) with default parameters for sequence-independent structural alignment. The TM-score was used to assess the structural similarity between the aligned proteins. Similarly, PF3D7_0619800 and PF3D7_0916400 were aligned with ScVps8 (PDB: 8QX8) and ScVps41 (PDB: 7ZU0), respectively.

To identify proteins structurally similar to PF3D7_0619800, PF3D7_0721000, and PF3D7_0916400, we performed a structural similarity search using Foldseek (https://search.foldseek.com/) against the AlphaFold/UniProt50 database with default parameters. Only hits with an *E* value < 0.00001 were considered.

### Figure generation

All the figures of the 3D structures presented in this study were generated using the open-source software ChimeraX (version 1.8) (87).

## Data Availability

The data supporting the findings of this study are available within the paper and are also available from the corresponding author upon request.

## SUPPLEMENTAL MATERIAL

Supplemental figures msphere.00287-25-s0001.pdf Figures S1 and S2.

Table S1 msphere.00287-25-s0002.xlsx Proteins identified in the Vps16 immunoprecipitation.

Table S2 msphere.00287-25-s0003.xlsx Blast analyses for the 3 uncharacterized proteins found in the Vps16 interactome.

ASM does not own the copyrights to Supplemental Material that may be linked to, or accessed through, an article. The authors have granted ASM a non-exclusive, world-wide license to publish the Supplemental Material files. Please contact the corresponding author directly for reuse.
