# Supplementary material for: The Plasmodium falciparum homolog of Vps16 interacts with the core members of the Vps-C tethering complex
Source: mSphere. 2025 Jul 8;10(7):e00287-25. doi: 10.1128/msphere.00287-25 (PMC12306165; doi:10.1128/msphere.00287-25)
Supplement: Supplemental figures — Figures S1 and S2. [file msphere.00287-25-s0001.pdf]

Fig. S1: PfVps16-2xFKBP-GFP is not mislocalized to the nucleus upon addition of rapamycin. (A) Live microscopy showing that incubating the PfVps16-2xFKBP-GFP+mislocalizer parasite line with 250 nM rapamycin for 24h or 48h does not result in the delocalization of the green signal to the nucleus. Arrows show overlapping foci between PfVps16-2xFKBP-GFP and the mislocalizer potentially outside of the nucleus. Scale bar represents 5µm. Blue: DAPI stained nucleus. RAPA: rapamycin. (Bi) Pearson's correlation analysis shows that the colocalization levels do not change between the DAPI and the PfVps16-2xFKBP-GFP (Bi) or the mCherry fused mislocalizer and PfVps16-2xFKBP-GFP (Bii). However, the colocalization between DAPI-stained nucleus and the mCherry-fused mislocalizer decreases upon the addition of rapamycin (Biii). Values represent the mean ± standard error. *P*-values were calculated using one-way ANOVA followed by a Tukey's multiple comparison test. NS=not significant. PfVps16-2xFKBP-GFP+3xNLS-FRB-BSD woRAPA, n=5. PfVps16-2xFKBP-GFP+3xNLS-FRB-BSD RAPA24, n=6. PfVps16-2xFKBP-GFP+3xNLS-FRB-BSD RAPA48, n=9.

Figure S2: AlphaFold3 structure prediction of PfVps3, PfVps11, PfVps16, PfVps18 and PfVps33. 3D structures are colour coded with predicted local-distance difference test (pLDDT) value.

Table S1: Mass spectrometry data from the two bioreplicates of the PfVps16 immunoprecipitations.

Table S2: Results from the BLAST analyses of PF3D7_0619800, PF3D7_0721000 and PF3D7_0916400

Fig S1

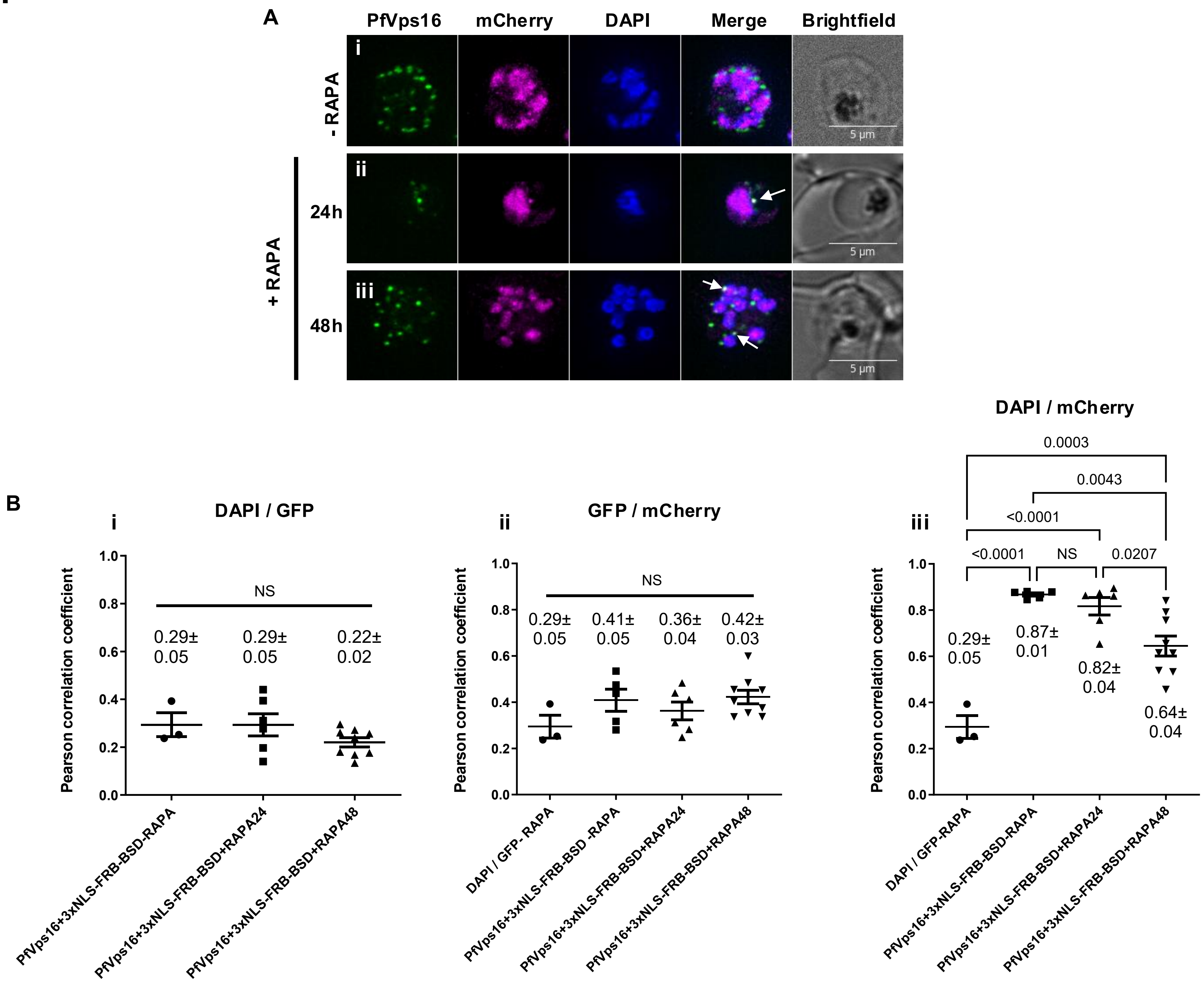
A
PfVps16
mCherry
DAPI
Merge
Brightfield
i
- RAPA
ii
iii
+ RAPA
24h
48h
5 µm
B
i
DAPI / GFP
Pearson correlation coefficient
NS
0.29± 0.05
0.29± 0.05
0.22± 0.02
PfVps16+3xNLS-FRB-BSD-RAPA
PfVps16+3xNLS-FRB-BSD+RAPA24
PfVps16+3xNLS-FRB-BSD+RAPA48
ii
GFP / mCherry
NS
0.29± 0.05
0.41± 0.05
0.36± 0.04
0.42± 0.03
DAPI / GFP- RAPA
PfVps16+3xNLS-FRB-BSD -RAPA
PfVps16+3xNLS-FRB-BSD+RAPA24
PfVps16+3xNLS-FRB-BSD+RAPA48
iii
DAPI / mCherry
0.0003
0.0043
<0.0001
<0.0001
NS
0.0207
0.29± 0.05
0.87± 0.01
0.82± 0.04
0.64± 0.04
DAPI / GFP-RAPA
PfVps16+3xNLS-FRB-BSD-RAPA
PfVps16+3xNLS-FRB-BSD+RAPA24
PfVps16+3xNLS-FRB-BSD+RAPA48

Fig S2

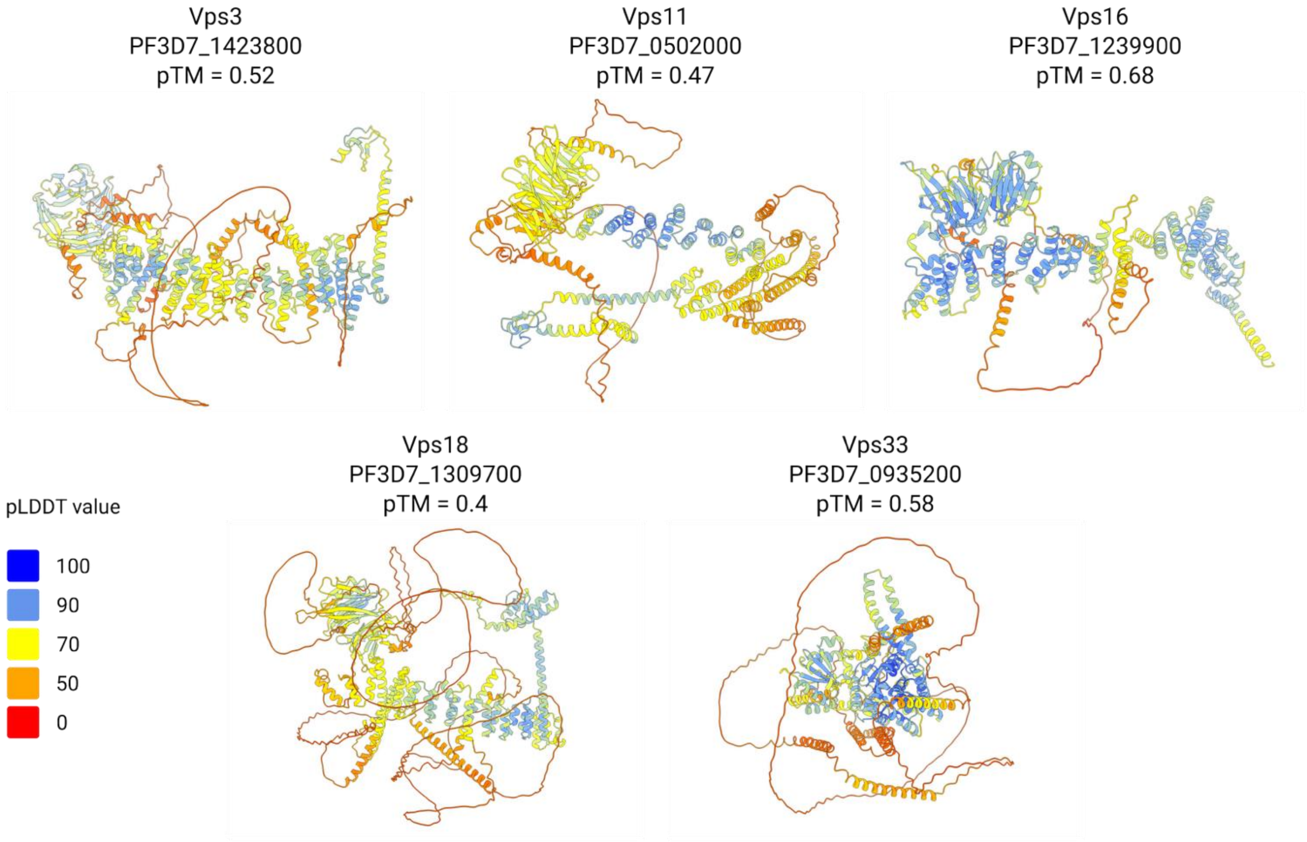
Vps3
PF3D7_1423800
pTM = 0.52
Vps11
PF3D7_0502000
pTM = 0.47
Vps16
PF3D7_1239900
pTM = 0.68
Vps18
PF3D7_1309700
pTM = 0.4
Vps33
PF3D7_0935200
pTM = 0.58
pLDDT value
100
90
70
50
0
